# Sodium channel subtype Na_v_1.3 is associated with increased hippocampal CA3 neuronal activity in early Alzheimer's Disease

**DOI:** 10.1002/alz70856_098112

**Published:** 2025-12-24

**Authors:** Jiabin Tang, Kishan Patel, Jiaxin Xiang, Hugh C Hemmings, Jimcy Platholi

**Affiliations:** ^1^ Weill Cornell Medicine, New York, NY, USA; ^2^ Weill Cornell Medicine, NEW YORK, NY, USA

## Abstract

**Background:**

Neuronal hyperexcitability is a prevalent feature in early stages of Alzheimer's Disease (AD), hastening cognitive decline and neurodegeneration. However, despite the immense clinical significance, the underlying molecular mechanisms are not fully understood. Variable expression of voltage‐gated sodium channel (Na_v_) subtypes in specific neuronal populations influence sensitivity of network excitability. Heterogeneity of Na_v_ expression also differs with cell maturity, as subtypes with distinct activation kinetics render developing neurons more susceptible to hyperexcitability when compared to established neurons. Interestingly, mature excitatory neurons, following injury or disease, augment their high‐frequency firing by reverting to an Na_v_ profile indicative of an earlier developmental window, but have yet to be explored in AD hyperexcitability. Here, we hypothesize that developmentally dominant Na_v_1.3 is re‐expressed in glutamatergic mossy fiber terminals and potentiates hippocampal CA3 hyperexcitability.

**Method:**

Subtype‐specific expression of Na_v_1.3 was quantified in mossy fiber terminals by immunogold labeling and electron microscopy from hippocampal tissue of 3‐mo (early stage) and 7‐mo (late stage) old male and female wildtype and 5xFAD mice. Real‐time measurements of neuronal activity from pyramidal CA3 neurons, synapsing with mossy terminals, were determined using fiber photometry in same mouse models and developmental stages. Neurophysiological properties of Na_v_1.3 were assayed using electrophysiology.

**Result:**

Here, we show that early AD mice have increased mossy terminal Na_v_1.3 labeling and hippocampal CA3 neuronal activity compared to wildtype or older AD mice. Higher neuronal activation was also found to be more prevalent in female mice at early stages of AD compared to male mice.

**Conclusion:**

Our findings identify a novel Na_v_ subtype (Na_v_1.3) re‐expressed in early stages of AD coinciding with sex dependent hippocampal CA3 hyperexcitability.

